# Integrating machine learning algorithms and single-cell analysis to identify gut microbiota-related macrophage biomarkers in atherosclerotic plaques

**DOI:** 10.3389/fcimb.2024.1395716

**Published:** 2024-04-23

**Authors:** Yin Ke, Jian Yue, Jiaming He, Guojing Liu

**Affiliations:** ^1^Department of Neurosurgery, The University-Town Hospital of Chongqing Medical University, Chongqing, China; ^2^Department of Nursing, The Maternal and Child Health Hospital of Yong Chuan, Chongqing, China; ^3^Department of Breast Surgery, Gaozhou People’s Hospital, Gaozhou, China; ^4^Institute of Life Sciences, Chongqing Medical University, Chongqing, China

**Keywords:** gut microbiota, macrophage, machine learning, atherosclerotic plaques, bioinformatics

## Abstract

**Objective:**

The relationship between macrophages and the gut microbiota in patients with atherosclerosis remains poorly defined, and effective biological markers are lacking. This study aims to elucidate the interplay between gut microbial communities and macrophages, and to identify biomarkers associated with the destabilization of atherosclerotic plaques. The goal is to enhance our understanding of the underlying molecular pathways and to pave new avenues for diagnostic approaches and therapeutic strategies in the disease.

**Methods:**

This study employed Weighted Gene Co-expression Network Analysis (WGCNA) and differential expression analysis on atherosclerosis datasets to identify macrophage-associated genes and quantify the correlation between these genes and gut microbiota gene sets. The Random Forest algorithm was utilized to pinpoint PLEK, IRF8, BTK, CCR1, and CD68 as gut microbiota-related macrophage genes, and a nomogram was constructed. Based on the top five genes, a Non-negative Matrix Factorization (NMF) algorithm was applied to construct gut microbiota-related macrophage clusters and analyze their potential biological alterations. Subsequent single-cell analyses were conducted to observe the expression patterns of the top five genes and the interactions between immune cells. Finally, the expression profiles of key molecules were validated using clinical samples from atherosclerosis patients.

**Results:**

Utilizing the Random Forest algorithm, we ultimately identified PLEK, IRF8, CD68, CCR1, and BTK as gut microbiota-associated macrophage genes that are upregulated in atherosclerotic plaques. A nomogram based on the expression of these five genes was constructed for use as an auxiliary tool in clinical diagnosis. Single-cell analysis confirmed the specific expression of gut microbiota-associated macrophage genes in macrophages. Clinical samples substantiated the high expression of PLEK in unstable atherosclerotic plaques.

**Conclusion:**

Gut microbiota-associated macrophage genes (PLEK, IRF8, CD68, CCR1, and BTK) may be implicated in the pathogenesis of atherosclerotic plaques and could serve as diagnostic markers to aid patients with atherosclerosis.

## 1 Introduction

Atherosclerosis is a stealthy vascular disease characterized by lipid accumulation and inflammation within the arterial intima, leading to plaque formation (Camare et al., 2017; Vergallo and Crea, 2020; Alkarithi et al., 2021). This process often progresses asymptomatically, yet when unstable plaques rupture, it can precipitate severe cardiovascular events such as myocardial infarction or stroke (Bailey et al., 2016). Rupture of unstable plaques releases necrotic tissue and structural components into the vasculature, activating the coagulation system and prompting platelet aggregation at the site of injury, forming thrombi (Mangge and Almer, 2019; Sterpetti, 2020). These thrombi may exacerbate arterial narrowing, obstruct blood flow, and cause myocardial ischemia due to oxygen and nutrient deprivation. If not promptly addressed, this ischemia can rapidly deteriorate, leading to life-threatening events. Thus, stabilizing atherosclerotic plaques and preventing their rupture is of paramount clinical importance for preventing acute cardiovascular incidents.

In the progression of atherosclerosis, macrophages within the arterial wall play a complex and pivotal role. They not only contribute to plaque formation by ingesting oxidized low-density lipoprotein (ox-LDL) and transforming into foam cells but also exacerbate local inflammation by releasing pro-inflammatory cytokines such as tumor necrosis factor-α (TNF-α) and interleukin-1β (IL-1β), attracting more immune cells and promoting plaque growth (Libby, 2021a). Macrophage death, particularly through the inflammatory cell death pathway of pyroptosis, releases factors that destabilize plaques, increasing the likelihood of rupture and thrombus formation (Wei et al., 2023). Additionally, macrophages differentiate into various phenotypes based on environmental signals, which play a decisive role in plaque stability and repair (Wolf and Ley, 2019; Libby, 2021b). They also participate in the reverse cholesterol transport, affecting the metabolic balance of plaques. Despite the crucial role of macrophages in atherosclerosis, the identification and clinical application of macrophage-related biomarkers remain challenging.

Recent research suggests a possible connection between the gut microbiota and atherosclerosis. Changes in the composition and metabolic activity of the gut microbiota, which affect host lipid metabolism, inflammation, and immune function, are thought to play a critical role in the formation and development of atherosclerosis. Notably, the gut bacterial metabolite trimethylamine N-oxide (TMAO), derived from the metabolism of choline, has been closely associated with the risk of atherosclerosis. Imbalances in the gut microbiota can also trigger systemic inflammatory responses and metabolic dysfunction, exacerbating the pathological process of atherosclerosis (Jonsson and Backhed, 2017; Ma et al., 2022). Modulating the gut microbiota with probiotics has opened new avenues for the treatment of atherosclerosis. However, the specific mechanisms by which the gut microbiota interacts with the host through its complex metabolic network to influence atherosclerosis require further scientific investigation.

With the ongoing progress of high-throughput sequencing, it’s become more common to investigate immune cells and gut microbiota characteristics with single-cell RNA sequencing and transcriptome analysis (Alcazar et al., 2022). In biomedicine, machine learning is increasingly applied to diagnose and predict disease outcomes (Danneskiold-Samsoe et al., 2019). These techniques analyze large data sets to uncover disease mechanisms and guide clinical decisions. This study seeks to identify macrophage genes closely tied to atherosclerotic plaques in relation to gut microbiota. We analyzed gene expression differences between normal carotid artery and atherosclerotic plaque samples, then identified key genes using machine learning algorithms.Finally, our single-cell and clinical sample analyses highlighted these genes’ pivotal role in plaque instability, offering novel insights for clinical approaches.

## 2 Materials and methods

### 2.1 Data collection

Bulk transcriptome datasets (GSE43292 and GSE120521) and single-cell datasets (GSE155512) were downloaded from the GEO database. GSE43292 contains 32 atheroma plaque and 32 macroscopically intact tissue samples, GSE120521 has four unstable and four stable plaque samples, and GSE155512 includes four atherosclerotic samples.

### 2.2 Differential expression analysis and WGCNA

Differential expression analysis was performed on the GSE43292 dataset for atheroma plaque and macroscopically intact tissue samples using the limma package, resulting in 188 upregulated genes in atherosclerosis plaques with criteria of |logFC| >0.8 and adjusted P-value <0.05. WGCNA algorithm was used to calculate gene significance (GS) and module membership (MM), confirming a key atherosclerosis module (blue module with 1115 genes) (Wang et al., 2023). Immune infiltration analysis was conducted using the deconvo_tme function from the IOBR package, and CIBERSORT was applied to WGCNA to calculate immune cell-related modules for M0, M1, and M2 macrophages, which were blue, red, and yellow, respectively (Wu et al., 2022). The union of genes from these three modules was defined as macrophage-related genes (a total of 2323 genes).

### 2.3 Pinpointing crucial macrophage genes

By integrating the 188 differentially expressed genes, the 1115 genes from the disease-related blue module, and the 2323 macrophage-related genes, a Venn diagram was constructed, ultimately yielding 139 genes. STRING database (https://cn.string-db.org/) was used to analyze these 139 genes, identifying key genes with a threshold score of 30 Degrees, and Cytoscape software was employed for visualizing protein-protein interactions (PPI) (Schroeder et al., 2013; Crosara et al., 2018). The cytoHubba plugin was then used to identify important genes, resulting in 24 significant genes. A random forest algorithm from the R package randomForest was applied to feature selection for these 24 genes, with the top 5 results defined as hub genes.

### 2.4 Collection of gut microbiota-related datasets

Gut microbiota-related gene sets were collected from GSEA (gsea-msigdb.org) using “Gut Microbiota” as the keyword (**Supplementary Table S1**). After scoring with the ssGSEA algorithm, correlation analysis with the five hub genes was performed to ultimately determine gut microbiota-related macrophage genes.

### 2.5 Construction of gut microbiota-related macrophage cluster

ConsensusClusterPlus package was used for consistency clustering analysis based on the expression of gut microbiota-related macrophage genes, ensuring the stability of the clustering assessment. The pheatmap package was utilized to visualize the expression differences of the five hub genes between groups, and PCA was visualized according to group and expression levels. GSEABase and GSVA packages were used to calculate immune scores for samples, visualizing differences in immune cell scores between groups and immune cell infiltration between high and low PLEK expression groups.

### 2.6 Construction of nomogram model for gut microbiota-related macrophage genes

The Nomogram model was constructed using the R package “rms,” based on the expression data of the TOP5 gut microbiota-related macrophage genes. The role of macrophage genes associated with gut microbiota in influencing disease outcomes was evaluated, and each gene was assigned a corresponding score. By summing these scores, a total score was obtained, which was used to assess the severity of atherosclerosis in patients.

### 2.7 Single-cell sequencing analysis

In the single-cell analysis phase, R packages Seurat and SingleR were used to process single-cell RNA sequencing data, with filtering criteria including retaining cells with counts greater than 200 and less than 10,000, and removing cells containing more than 20% mitochondrial genes or ribosomal genes. Data were then normalized, high-variance genes identified, and principal component analysis (PCA) performed for dimensionality reduction. Key principal components were determined through JackStraw analysis, and cell clustering analysis was conducted using the FindNeighbors and FindClusters functions from the “Seurat” package with a resolution of 1.2, followed by further analysis using t-SNE technology. The FindAllMarkers function from the Seurat package was used to identify differentially expressed genes, and cell annotation was based on the Human Primary Cell Atlas. Violin and heat maps were used to display the expression differences of atherosclerosis-related genes across different cell types. Additionally, macrophage sub-group analysis based on PLEK expression and GO and KEGG enrichment analyses were conducted to reveal changes in cell functions and signaling pathways. Finally, cell trajectories and intercellular communication networks were explored using the monocle and CellChat tools.

### 2.8 Clinical sample collection

After explaining our study to patients set for carotid endarterectomy, we got their approval and they signed consent forms. We collected four samples of stable plaques and five of unstable plaques. The study was approved by the ethics committee at The University-Town Hospital of Chongqing Medical University (Approval No: LL-202213).

### 2.9 Histology and immunohistochemistry

Fresh carotid atherosclerotic plaques were fixed overnight in 4% formaldehyde, then embedded in paraffin, and continuous 5-micron thick sections were obtained for subsequent experiments. The specific immunohistochemistry (IHC) protocol followed previously described methods (Jiang et al., 2019). We utilized the PLEK antibody from Proteintech, China. Based on the immunoreactive score method, the intensity of human atherosclerotic plaque tissue staining (protein expression) was scored range from 0-4, indicating negative staining to strong staining.

### 2.10 Western blot

For the specific Western Blot procedure, please refer to the methods previously described (Jiang et al., 2019). Ultimately, we assessed the expression levels of PLEK, P65, phospho-P65, IkBα, phospho- IkBα and GAPDH. All the aforementioned antibodies were sourced from Proteintech, China.

### 2.11 Cell culture

The RAW264.7 cell line was cultivated in DMEM (Gibco, USA), enriched with 10% FBS (Gibco, USA), and antibiotics penicillin (100 units/mL) and streptomycin (100 µg/mL). After washing with PBS, the cells were pre-treated with TNF-α (20 ng/mL) and LPS (100 ng/mL) from Abcam, USA, for 24 hours before proceeding with further experiments.

### 2.12 Lentiviral infection

Design primers, anneal and ligate them, and construct the sh-Plek plasmid with the sh sequence inserted at the AgeI and EcoRI restriction sites in the pLKO.1 vector. Revive normally growing 293T cells. The plasmid sequencing was successful, and lentivirus packaging was carried out at a ratio of 4:3:1 (sh-Plek, PsPAX.2, Pmd2.G) using Lipo3000 (Thermo Fisher, USA) as the transfection reagent. After 60 hours, collect the lentivirus using a 0.45 µm filter (Millipore, USA) and infect cells in six-well plates, with polybrene at a concentration of 8 µg/ml per milliliter. Change the medium 12 hours later, and passage the cells when they grow confluent, followed by antibiotic selection. The knockdown sequences are as follows: sh1- CCGG-GCTGGTTTCTAACAAGCTAGT-CTCGAG-ACTAGCTTGTTAGAAACCAGC-TTTTTT,sh2- CCGG-GGAGAACTCCAGTGATGATGA-CTCGAG-TCATCATCACTGGAGTTCTCC-TTTTTT,sh3- CCGG- GCCTACCTGCACTACTATGAT-CTCGAG- ATCATAGTAGTGCAGGTAGGC- TTTTTT.

### 2.13 Statistical analysis

In this study, R software version 4.1.2 and GraphPad Prism 7 were utilized for data analysis. We employed t-tests and Wilcoxon tests to assess the differences between the two groups. The significance levels are marked with asterisks: * for p-values below 0.05, ** for p-values less than 0.01, and *** for p-values under 0.001.

## 3 Results

### 3.1 Analysis of macrophage-related genes and immune infiltration in atherosclerotic plaques

**Figure 1:**
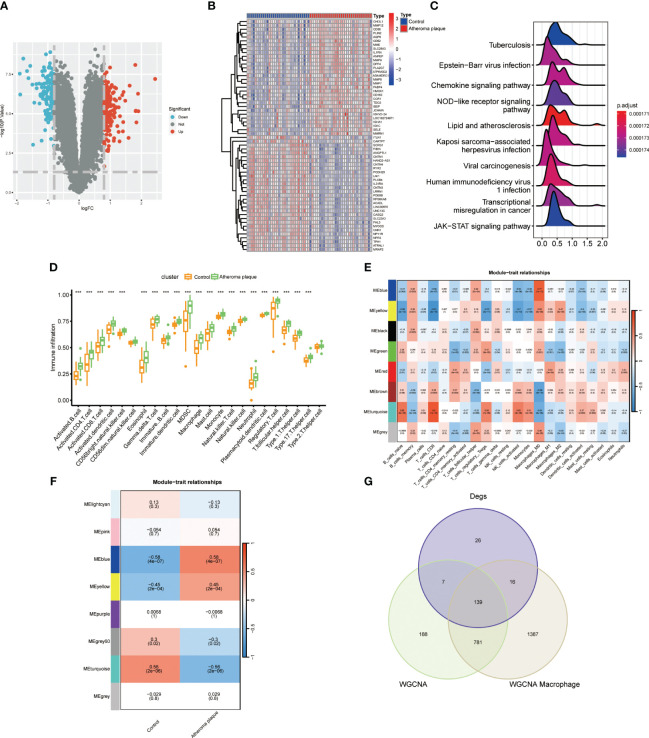
Identifyng macrophage genes linked to atherosclerotic plaques. **(A)** Volcano plot showing differentially expressed genes in atherosclerosis. **(B)** Heatmap displaying the top thirty specific differential genes between normal carotid artery and atherosclerotic plaque samples. **(C)** Signaling pathways highly related to atherosclerosis (X-axis: Represents the distribution range of the log2-transformed expression fold changes of core enrichment genes in enriched pathways). **(D)** Immune cell infiltration in normal carotid artery and atherosclerotic plaque samples. **(E)** WGCNA algorithm identifying key macrophage-related modules. **(F)** WGCNA analysis determining key modules in atherosclerosis. **(G)** VENN diagram identifying macrophage genes related to atherosclerotic plaques. (***P < 0.001).

We conducted a differential expression analysis on the GSE43292 dataset for atheroma plaque and macroscopically intact tissue samples (control group), identifying 142 downregulated and 188 upregulated genes (**Figure 1A**; **Supplementary Table S2**). Heatmap illustrates the top 30 genes with the most pronounced upregulation and downregulation (**Figure 1B**). Pathway enrichment analysis revealed significant enrichment in Neutrophil Extracellular Traps formation, Chemokine signaling pathway, and Lip and atherosclerosis (**Figure 1C**). Analysis of immune cell infiltration in atheroma plaque and macroscopically intact tissue samples showed a marked increase in inflammatory cell infiltration in the atheroma plaque group (**Figure 1D**). We employed the CIBERSORT algorithm to score immune cell-related modules, and the WGCNA algorithm to determine key modules related to M0, M1, and M2 macrophages, which were blue, red, and yellow, respectively, totaling 2323 genes (**Figure 1E**). Using the WGCNA algorithm, we identified a key module of 1115 genes in atherosclerosis (**Figure 1F**). A Venn diagram was used to identify 139 macrophage-related genes (**Figure 1G**).

### 3.2 Functional enrichment analysis of atherosclerotic plaque-related macrophage genes

**Figure 2:**
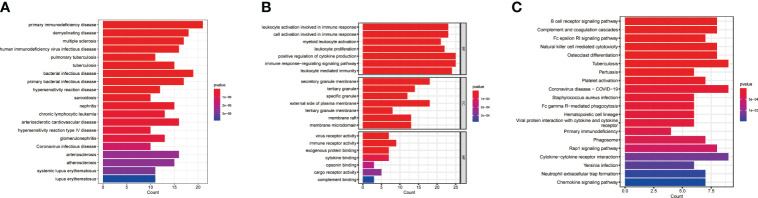
Pathway enrichment analysis. **(A)** DO enrichment analysis. **(B)** GO enrichment analysis. **(C)** KEGG enrichment analysis.

To explore the potential biological significance of macrophage-related genes, we performed DO/KEGG/GO enrichment analysis. The DO analysis significantly enriched in primary immunodeficiency disease and multiple diseases, the GO analysis significantly enriched in positive regulation of cytokine production and cytokine binding, and the KEGG analysis significantly enriched in cytokine-cytokine receptor interaction and primary immunodeficiency (**Figures 2A–C** and **Supplementary Table S3**).

### 3.3 Identification of gut microbiota-related core macrophage genes

**Figure 3:**
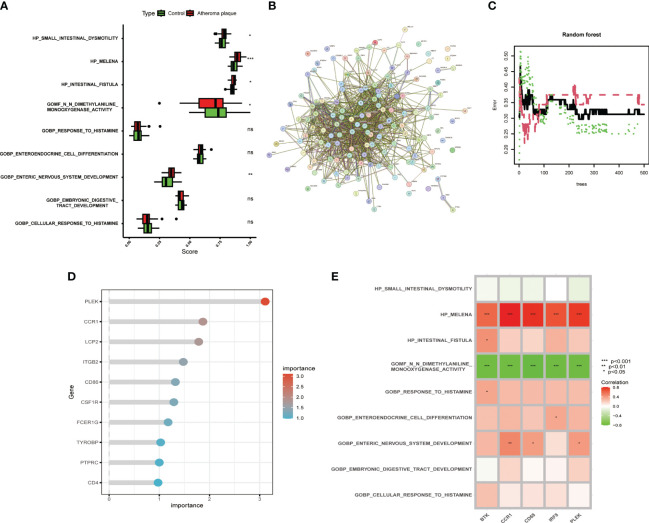
Identifying macrophage genes related to gut microbiota. **(A)** Boxplot showing changes in gut microbiota between normal carotid artery and atherosclerotic plaque samples. **(B)** Protein-protein interaction network of macrophage-related genes. **(C)** Random forest plot with red, green, and black dots representing atherosclerotic plaques, normal carotid artery, and all samples, respectively, with the x and y axes representing the number of trees and error rate. **(D)** Lollipop plot showing the top ten important genes. **(E)** Correlation analysis between the top five genes and gut microbiota-related gene sets. (*P < 0.05, **P < 0.01, ***P < 0.001).

The metabolic products of the gut microbiota can reach the vascular system through the bloodstream and affect macrophages, intervening in the development of atherosclerosis. However, more evidence is needed to deeply understand these complex relationships. Therefore, we compared the gut microbiota-related gene sets of atheroma plaque and macroscopically intact tissue samples. We found that the scores for HP_SMALL_INTESTINAL_DYSMOTILITY, HP_MELENA, HP_INTESTINAL_FISTULA, and GOBP_ENTERIC_NERVOUS_SYSTEM_DEVELOPMENT were significantly higher in the atheroma plaque group than in the control group,while the score for GOMF_N_N_DIMETHYLANILINE_MONOOXYGENASE_ACTIVITY was higher in the control group (**Figure 3A**). Subsequently, we performed protein-protein interaction (PPI) analysis on the 139 genes and visualized the results (**Figure 3B** and **Supplementary Table S4**). The random forest algorithm identified the top five genes (PLEK, IRF8, BTK, CCR1, and CD68), with PLEK being the most significant (**Figures 3C, D**). Finally, we conducted a correlation analysis between the top five genes and gut microbiota-related genes, showing that the top five genes were significantly positively correlated with HP_MELENA but significantly negatively correlated with GOBP_ENTERIC_NERVOUS_SYSTEM_DEVELOPMENT (**Figure 3E**). Ultimately, PLEK, IRF8, BTK, CCR1, and CD68 were confirmed as Gut Microbiota-Related Core Macrophage Genes.

### 3.4 Constructing a nomogram based on gut microbiota-related core macrophage genes

**Figure 4:**
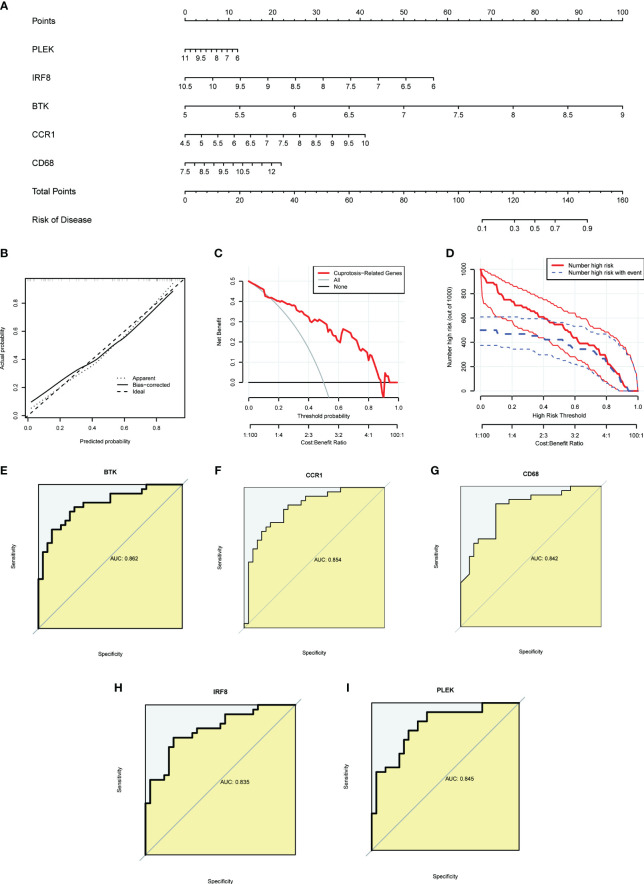
Constructing a nomogram related to gut microbiota macrophages to assess clinical value. **(A)** The nomogram demonstrates the prognostic value of the top five gut microbiota-related macrophage genes (PLEK, IRF8, BTK, CCR1, and CD68) for atherosclerosis patients. **(B)** Calibration curves to assess the degree of similarity between the predicted and true results of the gut microbiota-related macrophage nomogram. **(C)** Decision curve analysis to evaluate the sensitivity and specificity of the gut microbiota-related macrophage nomogram. **(D)** Clinical impact curve to assess the clinical impact of the gut microbiota-related macrophage nomogram at different thresholds. **(E–I)** ROC analysis results for the top five gut microbiota-related macrophage genes.

To provide targeted diagnostic assistance for atherosclerosis patients, we constructed a proprietary Nomogram model based on the expression of PLEK, IRF8, BTK, CCR1, and CD68 (**Figure 4A**). Calibration curve analysis revealed that the Nomogram model performed well in predictive accuracy, with its predictions closely matching the actual positive rates (**Figure 4B**). Decision curve analysis (DCA), clinical impact curve analysis (CICA), and ROC analysis further confirmed the significant clinical value of gut microbiota-related core macrophage genes in constructing the Nomogram model for atherosclerosis patients (**Figures 4C–I**). Analysis results in the GSE120521 dataset also showed that the expression levels of PLEK, IRF8, BTK, CCR1, and CD68 were significantly higher in the atheroma plaque group than in the control group (**Supplementary Figure S1**).

### 3.5 Construction and exploration of the biological characteristics of gut microbiota-related core macrophage clusters

**Figure 5:**
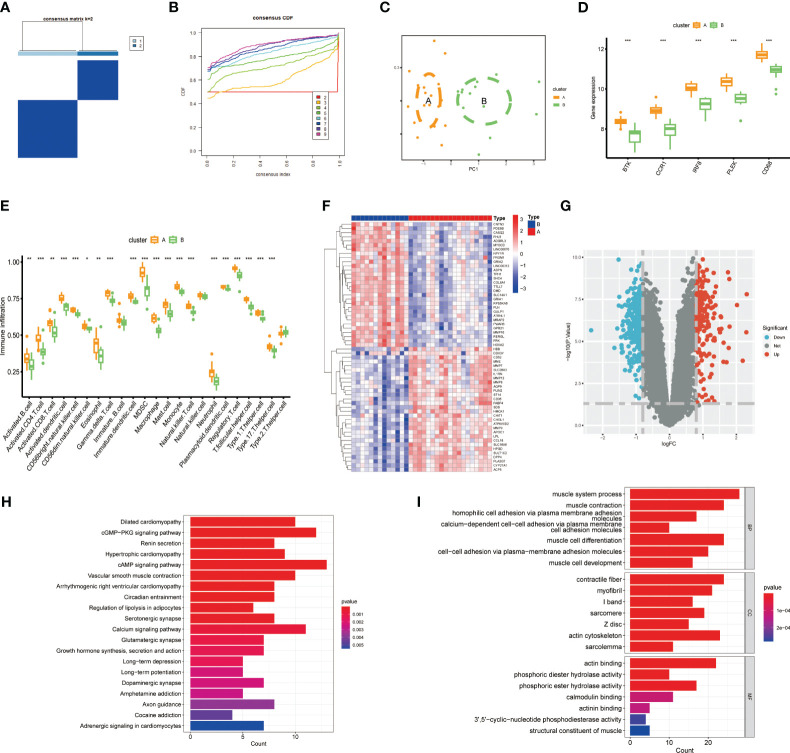
Constructing clusters of macrophages related to gut microbiota. **(A, B)** Clusters A and B were established using the NFM algorithm (consensus matrix k=2). **(C)** PCA analysis of Cluster A and Cluster B. **(D)** Expression of the top five genes between the two groups. **(E)** Expression of immune cells in Cluster A and Cluster B. **(F, G)** Results of differential analysis between Cluster A and Cluster B. **(H, I)** Pathway enrichment analysis of Cluster A-specifically expressed genes. (***P < 0.001).

To further investigate the potential role of gut microbiota-associated core macrophage genes in atherosclerosis, we applied unsupervised clustering methods based on the expression of PLEK, IRF8, BTK, CCR1, and CD68 to classify atherosclerosis patients into groups A and B (**Figures 5A, B**). The Principal Component Analysis (PCA) results indicated that macrophage genes associated with the gut microbiota could effectively categorize atherosclerosis patients into clusters A and cluster B (**Figure 5C**). Additionally, PLEK, IRF8, BTK, CCR1, and CD68 were more highly expressed in cluster A than in cluster B, and immune cell infiltration was also significantly higher in cluster A (**Figures 5D, E**). We then conducted a differential analysis between cluster A and cluster B and displayed the results using heatmaps and volcano plots (**Figures 5F, G**; **Supplementary Table S5**). Finally, we performed pathway enrichment analysis on the upregulated genes in cluster A, which were significantly enriched in pathways such as vascular smooth muscle contraction, regulation of lipolysis in adipocytes, muscle system process, muscle cell development, and muscle cell differentiation (**Figures 5H, I**).

### 3.6 Analyze the expression of gut microbiota-related macrophage genes in the atherosclerotic single-cell transcriptome

**Figure 6:**
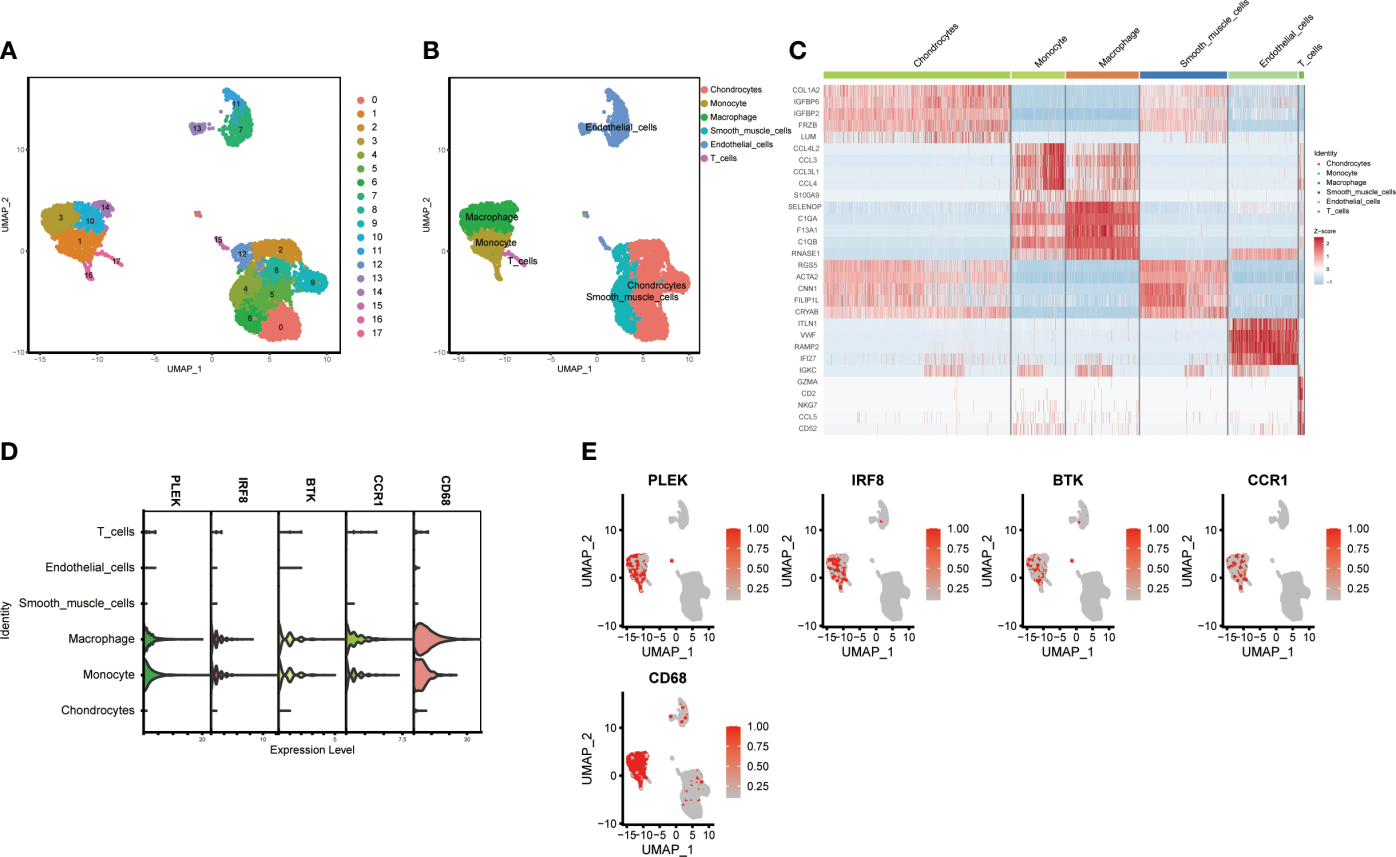
Single-cell subgroup annotation of atherosclerosis. **(A, B)** UMAP visualization of subgroup annotations. **(C)** Heatmap showing marker genes. **(D, E)** Expression of the top five gut microbiota-related macrophage genes in immune cells.

Using the “FindNeighbors” and “FindClusters” functions in the “Seurat” package for cell clustering analysis, we identified 17 cell clusters (**Figure 6A**). We used UMAP visualization to divide them into six cell populations: macrophages, monocytes, T cells, endothelial cells, chondrocytes, and smooth muscle cells, with heatmaps showing the marker genes for each subpopulation (**Figures 6B, C**). Finally, we found that PLEK, IRF8, BTK, CCR1, and CD68 were significantly expressed in monocytes and macrophages, further confirming the reliability of our previous transcriptome analysis (**Figures 6D, E**).

### 3.7 Investigating the effects of PLEK expression on macrophages

**Figure 7:**
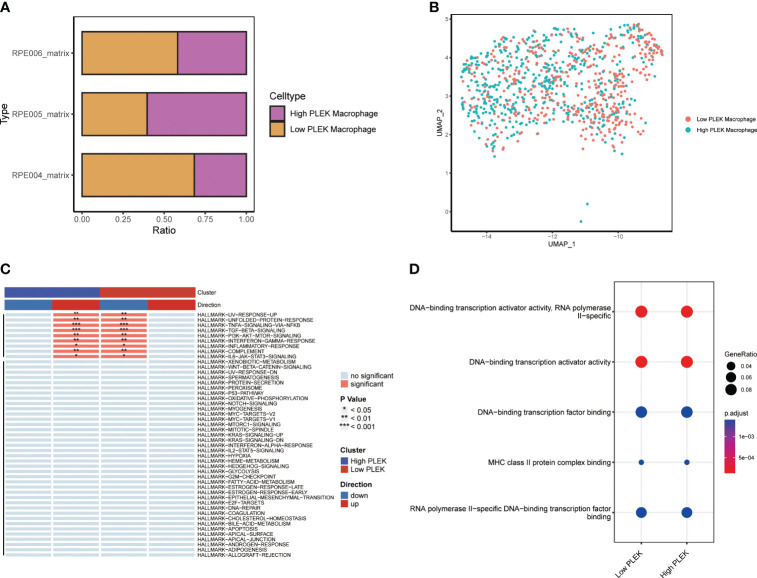
Single-cell level analysis of the impact of PLEK on macrophages. **(A, B)** Macrophages were divided into two groups based on PLEK expression and visualized using bar plots and UMAP. **(C)** Gene set enrichment analysis between high PLEK macrophage group and low PLEK macrophage group. **(D)** Pathway enrichment analysis between high PLEK macrophage group and low PLEK macrophage group.

Since the random forest tree results previously showed that PLEK was the most significant (**Figure 3D**), we divided macrophagesinto High PLEK Macrophage and Low PLEK Macrophage groups based on the median expression value of PLEK to analyze the biological changes between the two groups (**Figures 7A, B**). Using the irGSEA.score function for gene set analysis, we found that the TNFA-signaling-via-NFKB pathway was significantly upregulated in the High PLEK Macrophage group, while this pathway was significantly downregulated in the Low PLEK Macrophage group (**Figure 7C**). Subsequently, we performed pathway enrichment analysis on the differentially expressed genes between the PLEK high and low groups, which significantly enriched in DNA-binding transcription activator activity and DNA-binding transcription factor binding (**Figure 7D**).

### 3.8 Validate the expression of PLEK in atherosclerosis samples and investigate its impact on the NFκB pathway

**Figure 8:**
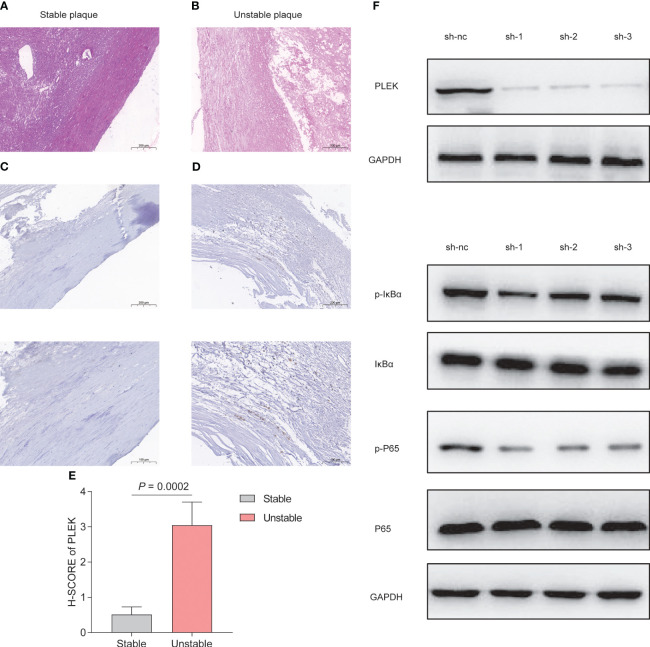
Experimental validation of PLEK. **(A, B)** HE staining showing stable atherosclerotic plaques and **(C)** expression of PLEK in stable and unstable atherosclerotic plaques. **(E)** H-SCORE of the two groups (*P* = 0.0002). **(F)** Validation of PLEK knockdown efficiency in RAW264.7 cells. **(G)** Western Blot detection of changes in p-P65, P65, p-IkBα, IkBα in RAW264.7 cells.

To validate the reliability of PLEK for diagnosing atherosclerosis patients, we collected samples from patients with atherosclerosis, including stable and unstable plaques (**Figures 8A, B**). PLEK expression was significantly higher in unstable plaques than in stable plaques (**Figures 8C–E**). Based on the results of **Figure 7C**, we found that PLEK might affect the NFκB signaling pathway. We stimulated RAW264.7 cells and PLEK-knockdown RAW264.7 cells with LPS and TNF-α to observe whether PLEK would influence the atherosclerosis process through the NFκB signaling pathway (**Figure 8F**). The results indicated that after the knockdown of PLEK, P65 and IκBα remained unchanged, while there was a significant downregulation of p-P65 expression (**Figure 8G**).

## 4 Discussion

Atherosclerosis, a complex and multifaceted disease, is characterized by the accumulation of lipids, inflammation, and fibrous thickening within the arterial walls, leading to vascular stenosis or occlusion and a heightened risk of cardiovascular events. Dysregulation of lipid metabolism, endothelial dysfunction, and infiltration of inflammatory cells synergistically contribute to plaque development. The rupture of an unstable plaque can precipitate thrombosis and trigger acute cardiovascular incidents. Current therapeutic approaches for atherosclerosis encompass pharmacological interventions aimed at mitigating plaque progression and enhancing vascular function. Early diagnosis is pivotal for effective prevention and management of this disease. Our research endeavors to identify and validate early biomarkers of atherosclerosis, facilitating timely interventions and improving patient outcomes. Macrophages, with their dualistic role, are instrumental in the pathogenesis of atherosclerosis, modulating the inflammatory milieu of plaques and thereby influencing disease progression (Tabas and Bornfeldt, 2020). The gut microbiota’s intricate relationship with atherosclerosis is increasingly recognized, with dysbiosis potentially exacerbating lipid metabolism disorders and amplifying inflammatory responses. The interplay between the gut microbiota and macrophages is particularly intriguing, yet the specific contributions of gut microbiota-associated macrophage genes to atherosclerosis remain to be fully elucidated.Our study delves into the potential of gut microbiota and macrophages as significant factors in atherosclerosis, aiming to uncover novel insights into the disease’s pathophysiology. By identifying and analyzing key genes associated with the gut microbiota and macrophage activity, we seek to unravel the underlying biological mechanisms and chart a course for innovative diagnostic and therapeutic strategies.

In our comparative analysis of gut microbiota-related gene set activities between normal carotid artery and atherosclerotic plaque samples, we discerned a pronounced elevation in the activities of HP_SMALL_INTESTINAL_DYSMOTILITY, HP_MELENA, HP_INTESTINAL_FISTULA, and GOBP_ENTERIC_NERVOUS_SYSTEM_DEVELOPMENT in the cohort with atherosclerotic plaque samples. This finding is corroborated by research highlighting the intimate connection between the progression of non-alcoholic fatty liver disease and disruptions in gut microbiota, as well as intestinal dysfunction (Leung et al., 2016), underscoring the burgeoning research potential in elucidating the interplay between gut microbiota and atherosclerosis. Conversely, the normal carotid artery group exhibited a notable increase in N_N_DIMETHYLANILINE_MONOOXYGENASE_ACTIVITY. Prior studies have posited that plasma TMAO levels do not correlate with the incidence of atherosclerosis but are markedly positively associated with unstable plaques (Koay et al., 2021). This discrepancy may suggest that TMAO within the arterial intima possesses distinct biological implications compared to its plasma counterpart, warranting further exploration into the nuanced roles of TMAO in the arterial wall’s pathology.

In recent years, the Nomogram model has emerged as a reliable clinical diagnostic tool, demonstrating exceptional predictive accuracy and offering personalized diagnostic strategies for patients (Tong et al., 2023). Leveraging machine learning algorithms, we have identified PLEK, IRF8, BTK, CCR1, and CD68 as evaluative markers to assist in the diagnosis of atherosclerosis. Studies have reported a significant upregulation of PLEK in ulcerative colitis and rheumatoid arthritis (Chen et al., 2020). Sequence variants near the IRF8 gene have been implicated as key risk factors for inflammatory bowel disease and multiple sclerosis (Salem et al., 2020). BTK inhibitors, approved for the treatment of leukemia and lymphoma (Brullo et al., 2021), raise intriguing questions about their potential role in atherosclerosis patients. Our research has uncovered the specific expression of CCR1 in macrophages, paralleling the observed increase in CCR1 expression on inflammatory cells in patients with severe chronic obstructive pulmonary disease (COPD) (Nakano et al., 2018). CD68, a common macrophage marker, is well-documented in its association with a variety of inflammatory diseases, further substantiating its relevance in the context of atherosclerosis. At the single-cell level, we classified macrophage subpopulations based on the expression levels of the PLEK, dividing them into high PLEK-expression macrophages and low PLEK-expression macrophages around the median value. This stratification revealed a concomitant upregulation of TNFα-signaling-via-NFKB, IFNG response, and inflammatory response pathways in macrophages with heightened PLEK expression. Given the established role of macrophages in orchestrating inflammatory responses, which are critical in the pathophysiology of numerous immune-mediated conditions (Koelwyn et al., 2018), our bioinformatics findings lead us to hypothesize that increased PLEK expression on macrophages may potentiate their inflammatory profile.

In summary, this investigation harnesses a comprehensive suite of bioinformatics tools to uncover the intricate connections between the gut microbiota, macrophage function, and the pathogenesis of atherosclerosis, thereby contributing fresh perspectives to our understanding of this complex disease. Despite these advancements, our study acknowledges limitations, such as the reliance on existing sequencing data, the finite scope of human sample cohorts, and the imperative for more profound exploration into the underlying biological mechanisms.

## 5 Conclusion

In summary, our study comprehensively examined the relationship between gut microbiota-associated macrophage genes and atherosclerosis, leading to the identification of pivotal genes. Subsequently, these key genes were subjected to an integrated analysis alongside immune cell dynamics, gut microbiota activity, and single-cell profiling. Ultimately, PLEK was revealed as a potential driver in the formation of unstable atherosclerotic plaques.

## Data availability statement

The original contributions presented in the study are included in the article/**Supplementary Material**. Further inquiries can be directed to the corresponding author.

## Ethics statement

The studies involving humans were approved by the University-Town Hospital of Chongqing Medical University LL-202213. The studies were conducted in accordance with the local legislation and institutional requirements. The participants provided their written informed consent to participate in this study.

## Author contributions

YK: Writing – original draft, Visualization, Validation, Formal analysis, Conceptualization. JY: Writing – original draft, Supervision, Methodology, Conceptualization. HJ: Writing – review & editing, Methodology, Conceptualization. GL: Writing – review & editing, Supervision, Investigation, Conceptualization.

## Supplementary material

Supplementary Figure 1 GSE120521 validation of the expression of PLEK, IRF8, BTK, CCR1, and CD68.
