# Supplementary figures and images for: Integrating machine learning algorithms and single-cell analysis to identify gut microbiota-related macrophage biomarkers in atherosclerotic plaques
Source: Front Cell Infect Microbiol. 2024 Apr 23;14:1395716. doi: 10.3389/fcimb.2024.1395716 (PMC11074432; doi:10.3389/fcimb.2024.1395716)

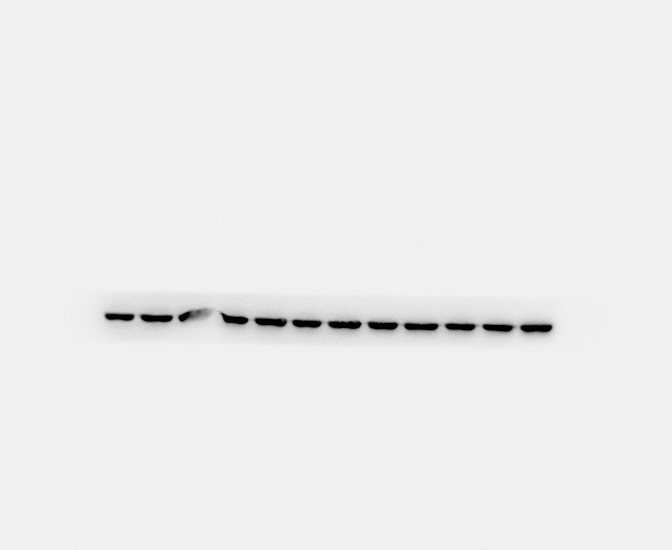

Supplement: Supplementary file 1 [file DataSheet_1.zip › west blot/Fig8E GAPDH.tif]

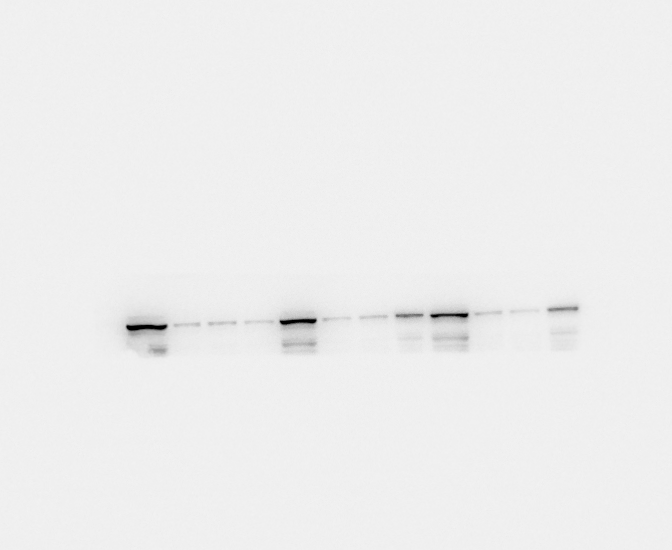

Supplement: Supplementary file 1 [file DataSheet_1.zip › west blot/Fig8E.PLEK.tif]

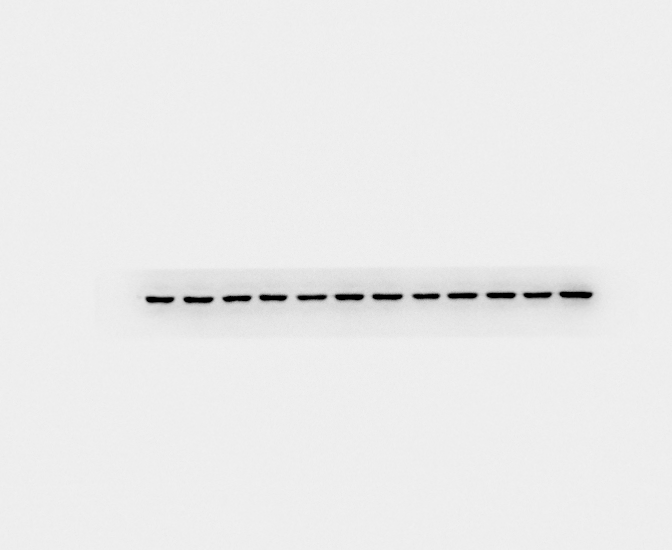

Supplement: Supplementary file 1 [file DataSheet_1.zip › west blot/Fig8F.GAPDH.tif]

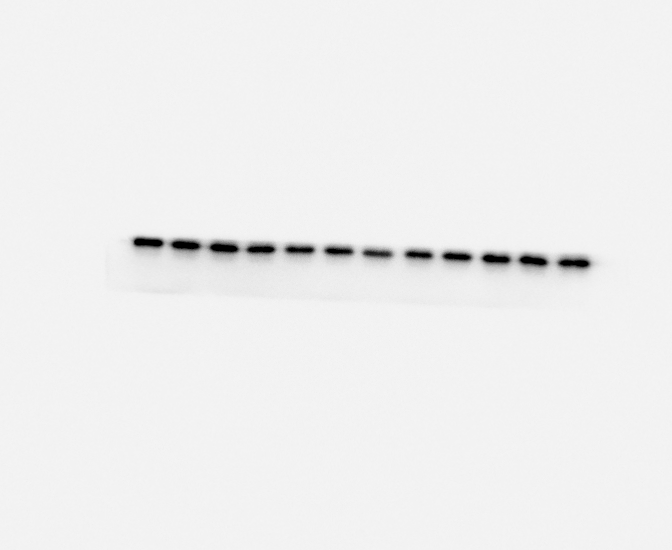

Supplement: Supplementary file 1 [file DataSheet_1.zip › west blot/Fig8F.ikbα.tif]

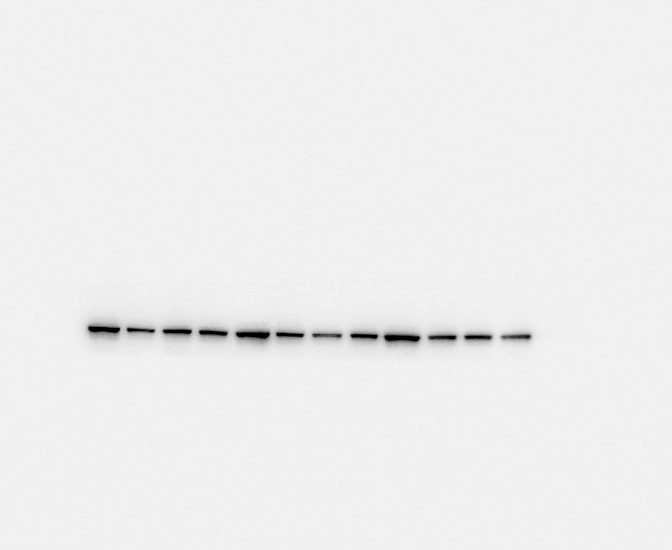

Supplement: Supplementary file 1 [file DataSheet_1.zip › west blot/Fig8F.p-ikbα.tif]

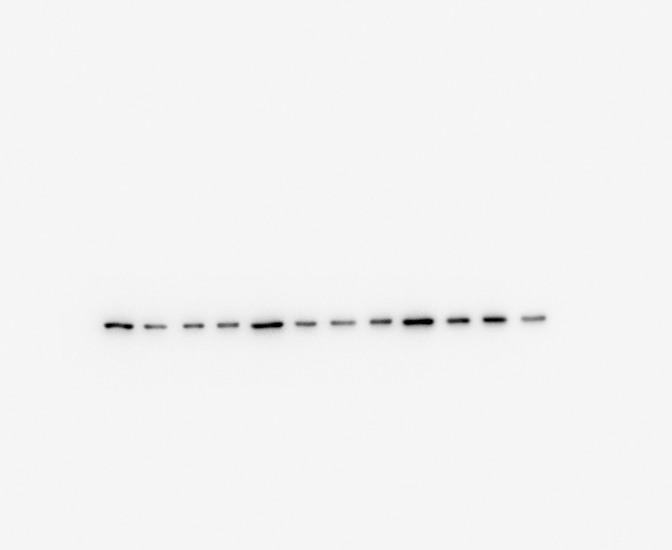

Supplement: Supplementary file 1 [file DataSheet_1.zip › west blot/Fig8F.p-p65.tif]

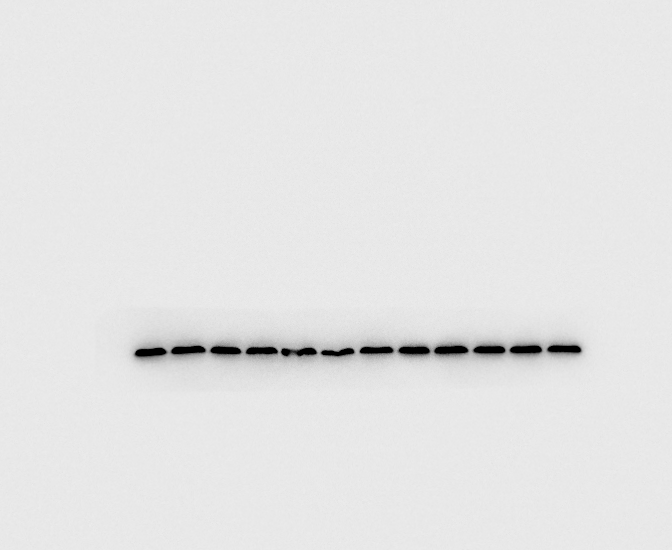

Supplement: Supplementary file 1 [file DataSheet_1.zip › west blot/Fig8F.p65.tif]

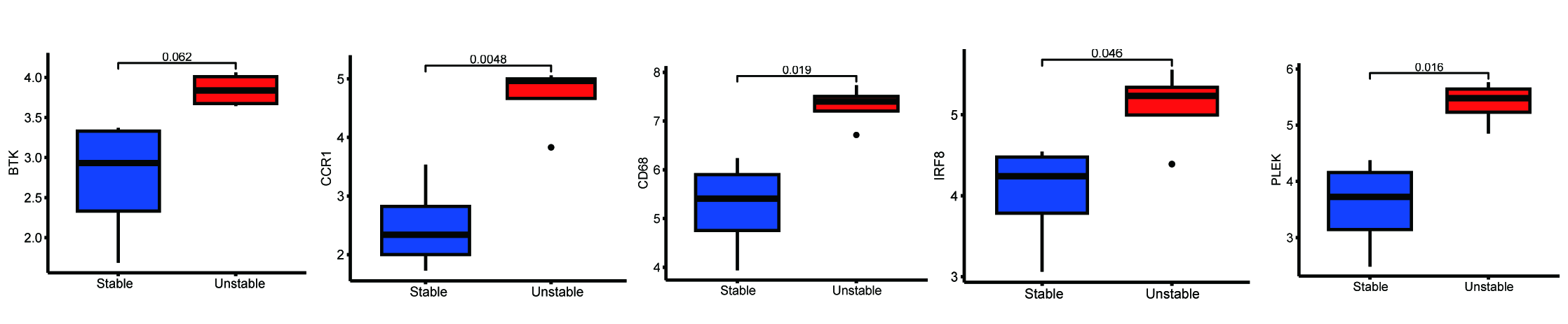

Supplement: Supplementary Figure 1 — GSE120521 validation of the expression of PLEK, IRF8, BTK, CCR1, and CD68. [file Image_1.tif]
